# Increased Expression of AbcA Efflux Pump Accelerated Resistance Development from Tolerance to Resistance Against Oxacillin in *Staphylococcus aureus*

**DOI:** 10.3390/microorganisms13051140

**Published:** 2025-05-16

**Authors:** Xiaohui Yu, Miaomiao Liu, Pilong Liu, Zehua Hao, Lili Zhao, Xin Zhao

**Affiliations:** 1 Hebei Key Lab of Laboratory Animal Science, Department of Laboratory Animal Science, Hebei Medical University, Shijiazhuang 050017, China; 19201782@hebmu.edu.cn; 2 College of Animal Science and Technology, Northwest A&F University, Xianyang 712100, China; 17863801579@163.com (M.L.); liupilong_2008@hotmail.com (P.L.); haozehua1016@163.com (Z.H.); 2015120006@nwafu.edu.cn (L.Z.); 3 Department of Animal Science, McGill University, Montréal, QC H9X 3V9, Canada

**Keywords:** *Staphylococcus aureus*, AbcA efflux pump, tolerance, resistance, resistance development

## Abstract

Bacterial tolerance, especially in *Staphylococcus aureus* (*S. aureus*), may arise under intermittent antibiotic regimens and act as a stepping stone toward resistance development. However, the transition from tolerance to resistance and its contributing factors remain poorly understood. This study explores the role of the efflux pump gene *abcA* in this process. *abcA* mutants (overexpression, knockout, and complementation) were constructed via homologous recombination. These strains were subjected to 21 cycles of intermittent exposure to oxacillin at 20× MIC, and the resistance evolution was monitored. Spontaneous mutation frequencies and survival abilities in these mutants were also measured to determine their involvement in resistance development. The *abcA* overexpression mutant exhibited a faster development of resistance compared to the wildtype strain. Conversely, the *abcA* knockout mutant maintained susceptibility to oxacillin, with no significant changes in the relative MIC. Increased mutation frequency and enhanced survival were observed in the overexpression strain, whereas both were reduced in the knockout. *abcA* overexpression significantly accelerated the development of oxacillin resistance in *S. aureus* by promoting spontaneous mutations and bacterial survival. Disrupting *abcA* may offer a novel strategy to prevent the evolution of antibiotic resistance.

## 1. Introduction

Antimicrobial resistance (AMR) is rapidly spreading among opportunistic pathogens, posing a growing threat to global public health. Predictive models estimate that, in 2019 alone, AMR contributed to around 4.95 million deaths (range 3.62–6.57 million) [1], and the annual death toll is expected to soar to 10 million by 2050 [2]. Among the various pathogens contributing to AMR, *Staphylococcus aureus* (*S. aureus*) stands out due to its role in severe infections such as endocarditis, sepsis, and pyemia. In 2019, *S. aureus* accounted for approximately 26.1% of all AMR-related deaths, among the six key pathogens identified by the World Health Organization (WHO) [1]. The rise of β-lactam antibiotic resistance, including resistance to methicillin, oxacillin, and penicillin, underscores the urgency of this issue. These antibiotics are primary treatments for severe infections, yet over 70% of AMR-related mortalities are attributed to resistance against them and fluoroquinolones. In response, the WHO has identified methicillin-resistant *Staphylococcus aureus* MRSA as one of the critical pathogens requiring urgent antibiotic development efforts [3,4]. A thorough understanding of the evolutionary mechanisms behind β-lactam resistance is critical to combating the escalating AMR challenge by disrupting the process of resistance development.

Bacteria adopt two primary strategies to develop resistance. Under sustained exposure to low levels of antibiotics, especially at sub-lethal concentrations, pathogens rapidly acquire antibiotic resistance [5,6,7,8]. This resistance may arise through multiple strategies, including enzymatic breakdown of the drug, structural alterations of antibiotic targets, or impaired intracellular accumulation due to limited membrane permeability or activation of efflux systems [9]. By contrast, exposure to transiently elevated antibiotic levels—often exceeding the Minimum Inhibitory Concentration (MIC)—can select for tolerant phenotypes, enabling bacteria to persist temporarily under lethal drug concentrations [10,11]. For instance, dormancy-induced tolerance has been found to protect bacteria from the attack of various antibiotics that necessitate bacterial growth to be effective [12]. Both genetic and environmental factors can induce tolerance [13,14]. Despite substantial progress in resistance research, interest has only recently shifted to understanding how tolerance contributes to resistance, especially in persistent infection settings [15,16,17,18]. Studies have shown that bacterial tolerance can rapidly evolve into resistance, as observed in *Escherichia coli* (*E. coli*) and *S. aureus* [19,20,21]. The rapid evolution from tolerance to resistance indicates that certain factors within tolerance are promoting this resistance acquisition. However, the specific contributors that facilitate the transition from tolerance to resistance are still not inadequately elucidated.

Efflux pumps, ubiquitous transporter proteins found in both prokaryotic and eukaryotic cells, are recognized as a key resistance mechanism by extruding antibiotics from bacterial cells [22]. Although resistance mechanisms have been extensively studied, how efflux pumps influence the progression from bacterial tolerance to full resistance remains insufficiently understood. Given that tolerance is known to accelerate the subsequent evolution of resistance, as documented by Levin-Reisman et al. [19], the observed upregulation of multiple multidrug efflux genes in tolerant cells suggests a potential role for efflux pumps as contributors to the progression from tolerance to resistance [12]. Our previous work revealed that the NorA efflux pump promoted resistance development against ciprofloxacin [16]. However, the involvement of other efflux pumps, such as the AbcA pump from the ATP-binding cassette family [23], remain unclear.

AbcA is important in bacterial physiology, such as antibiotic resistance and virulence. AbcA can extrude β-lactam drugs, including oxacillin, to confer β-lactam resistance [24,25]. AbcA also contributes to *S. aureus* virulence by secreting phenol-soluble modulins [26,27]. A recent study highlighted AbcA’s participation in tolerance formation, where tolerant cell levels decreased in an *abcA* knockout mutant under exposure to 25× the MIC of nafcillin [28]. However, the specific involvement of AbcA in mediating the shift from a tolerant to a resistant phenotype has not been investigated.

This study aimed to elucidate the role of the AbcA efflux pump in the development of antibiotic resistance, specifically through the lens of bacterial tolerance. We previously conducted in vitro evolutional experiments with the wildtype Newman. The resultant oxacillin tolerance strain OXA.C13.T5 was used in this study to investigate the role of AbcA in the development of resistance from tolerance. This was achieved through engineering overexpression, knockout, and complement mutants of *abcA* and monitoring resistance development in these mutants during evolutionary experiments. Our second objective was to uncover how AbcA influenced—if it had an effect—this resistance development by assessing the mutation and survival rates of these *abcA* mutants.

## 2. Materials and Methods

### 2.1. Bacterial Strains and Cultivation Methods

The four tolerant *S. aureus* strains (Table S1) used in this study were derived from the wildtype Newman strain. These tolerant strains were developed through periodic 5 h exposures to 20× MIC concentrations of different antibiotics—oxacillin, imipenem, flucloxacillin, and meropenem—as detailed in the evolutionary adaptation procedure in the Supplementary Materials. *S. aureus* strains were grown either in a TSB liquid medium with shaking or on solid TSB agar plates at 37 °C. The cultivation of *E. coli* was performed using Luria-Bertani (LB) medium under comparable conditions. For long-term preservation, bacterial cultures were combined with glycerol to a final concentration of 50% *v*/*v* and stored at −80 °C.

### 2.2. Construction of AbcA Mutants

The *abcA* mutants (overexpression and knockout) were created according to the protocol established by Li et al. (2020) [29]. The wildtype Newman strain, with unaltered *abcA* expression, served as a control. Overexpression was achieved by introducing the pSE1 vector, which carries the *abcA* gene, into the Newman strain. For the knockout study, *abcA* was knocked out in the OXA.C13.T5 strains using the pKZ2 vector, resulting in the ∆*abcA* mutant. Restoration of *abcA* in the knockout mutant was accomplished using the shuttle plasmids pSC1 containing the *abcA* gene. More detailed mutant-construction procedures, including vector preparation and the primers used (Table S2), are shown in the Supplementary Materials.

### 2.3. In Vitro Adaptive Evolution Experiment

The in vitro adaptive evolution strategy was adapted from a previously established protocol [11] and our prior methodology, involving repeated high-dose (20× MIC) exposure of the *abcA* mutants (overexpression, knockout, and complemented strains) to antibiotics. This cyclic process was performed over 7 to 21 rounds until resistance was detected. Each evolution cycle included the following three phases: antibiotic challenge, drug withdrawal, and recovery growth. Specifically, 20 μL of overnight culture was transferred into 2 mL of TSB containing 20× MIC of the corresponding antibiotic. The culture was incubated at 37 °C with agitation for 5 h, followed by two PBS washes and centrifugation at 1500× *g* for 20 min to eliminate residual antibiotic. The resulting pellet was resuspended in 2 mL of fresh TSB and cultured overnight. Half of the overnight culture was used to initiate the next evolution cycle, while the remainder—after verifying purity based on colony morphology on TSA—was cryopreserved at −80 °C for downstream analyses.

### 2.4. Mutation Rate Estimation

Mutation frequency was measured following the procedure described by El Meouche et al. [30], with minor adaptations. Frozen *S. aureus* stocks were streaked on Mueller–Hinton II (MH2) agar to obtain isolated colonies. Three representative colonies with uniform morphology were selected per strain and inoculated into TSB. After 24 h of incubation, the cultures were serially diluted (10^7^-fold) to reduce the potential impact of pre-existing mutants from the stationary phase. Each colony-derived dilution was used to establish eight replicate cultures, which were incubated overnight at 37 °C. To estimate population density, 5 μL from each replicate was diluted (10^7^–10^8^-fold) and plated onto plain TSB agar. In parallel, 100 μL aliquots were spread onto rifampicin-supplemented agar (100 mg/L). Non-selective plates were incubated for 24 h, and selective plates for 48–72 h, both at 37 °C. Mutation frequency was calculated as the ratio of colony-forming units (CFUs) on the rifampicin plates to those on the drug-free plates.

### 2.5. Survival Ability Assessment Under Antibiotic Pressure

Overnight *S. aureus* cultures originating from a single colony were diluted 1:100 into fresh TSB. Antibiotics were then added at concentrations equivalent to 20× MIC. At defined time intervals (12, 24, and 36 h), 500 μL aliquots were withdrawn, serially diluted, and spread onto TSB agar. To eliminate residual antibiotic interference, cells were washed with PBS prior to plating. Plates were incubated for a minimum of 48 h to allow for visible colony formation. Each experiment was performed independently at least three times, with multiple technical replicates, to ensure the robustness and reproducibility of the findings.

### 2.6. Statistical Analysis

Statistical analyses were conducted using GraphPad Prism (v9.0.0, Mac OS). Statistical significance was assessed via one-way ANOVA, followed by LSD or Dunnett’s T3 multiple comparisons test. Two-tailed *p* values less than 0.05 were interpreted as statistically significant (*), while values below 0.01 were considered highly significant (**).

## 3. Results

### 3.1. Tolerant Strain OXA.C13.T5 Developed Resistance Faster Than the Wildtype Strain Newman

**Figure 1:**
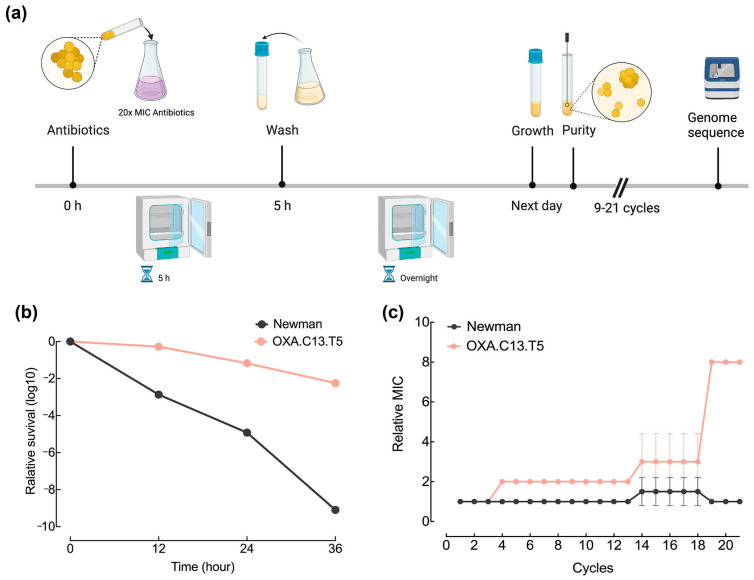
Tolerant strain OXA.C13.T5 developed oxacillin resistance faster than wildtype Newman. (**a**) Schematic presentation of intermittent oxacillin challenge cycles for the tolerant strain OXA.C13.T5 and the wildtype Newman strain; (**b**) Killing kinetics over a 36 h treatment period with 20× MIC oxacillin; viable cells were quantified at 12 h intervals via colony counts. (**c**) MIC progression in evolved strains, compared to their respective baseline MICs at cycle 0.

We previously acquired four tolerant strains by the intermittent exposure of the wildtype strain Newman to four β-lactam antibiotics (oxacillin, imipenem, flucloxacillin, and meropenem) (Table S1). All four tolerant strains possessed mutations in genes such as *gdpp* encoding c-di-AMP phosphodiesterase for OXA.C13.T5, *pth* encoding peptidyl-tRNA hydrolase for IMI.C9.T5, *545* encoding unknown 545 for FLUC.C20.T5, and *map* encoding MHC class II analog protein for FLUC.C20.T5. The *gdpp* mutation was responsible for the tolerance phenotype of the OXA.C13.T5 strain to oxacillin, as the OXA.C13.T5 strain exhibited a characteristic tolerance phenotype with delayed killing under 20× MIC of oxacillin (Figure 1b). On the other hand, a similar killing time was observed between the *gdpp*-restored OXA.C13.T5 strain and the Newman strain (unpublished data). To illustrate whether the tolerance background impacted the development from tolerance to resistance, the wildtype strain Newman and the tolerant strain OXA.C13.T5 were intermitted challenged with 20× MIC oxacillin for 5 h and continued for 21 cycles (Figure 1a). Changes in the MIC during exposure indicated that the tolerant strain OXA.C13.T5 became resistant against oxacillin at the 19th cycle, while the Newman strain remained susceptible to oxacillin until the 21st cycle (Figure 1c), suggesting that the existence of some factors in tolerant strains contributed to resistance development.

### 3.2. Increased Expression of Efflux Pump AbcA Across Oxacillin Tolerant Strains

**Figure 2:**
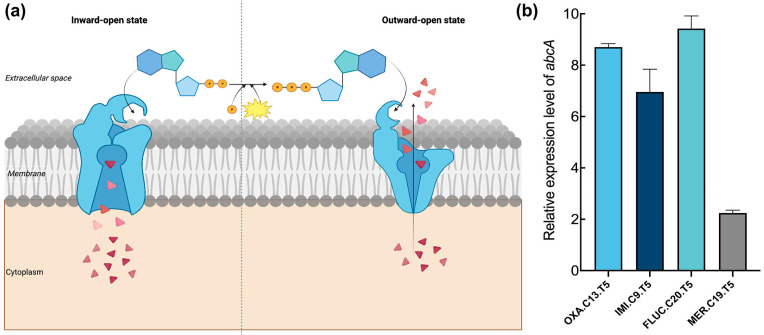
Expression of *abcA* in tolerant strains. (**a**) ABC efflux pumps extrude antibiotics by changing inward-open state to outward-open state driven by ATP. The left part is the inward-open state, which changes into the outward-open state (right part) after acquiring ATP. As the arrow shows, this transformation changes pumps antibiotics out from the inside to the outside. Red triangles represent antibiotic molecules, orange circles represent ATP, and the yellow burst indicates the energy release that drives the conformational change. (**b**) The relative expression level of *abcA*. Each tolerant strain’s expression is relative to the expression level of the wildtype strain.

It is well known that ABC transporters contribute to bacterial pathogenesis and virulence, as well as multidrug resistance, through exporting xenobiotics [31]. We investigated whether ABC transporters contribute to the transition from a tolerant phenotype to a resistant one. Therefore, the expression level of *abcA*, which encodes an ATP-binding cassette (ABC) efflux transporter operating via ATP-driven conformational cycling between inward- and outward-facing states (Figure 2a), was quantified in four tolerant strains using real-time PCR. An elevated relative expression level of *abcA* was observed in all selected tolerant strains, with increases of over 6-fold for OXA.C13.T5, IMI.C9.T5, and FLUC.C20.T5 strains relative to Newman (Figure 2b). Although FLUC.C20.T5 showed the least change, its relative expression level of *abcA* still increased two-fold. The increased expression of *abcA* across the tolerant strains suggests that it may play an important role in promoting the development of resistance from tolerance.

### 3.3. Increased Expression of AbcA Accelerated Resistance Development Against Oxacillin

**Figure 3:**
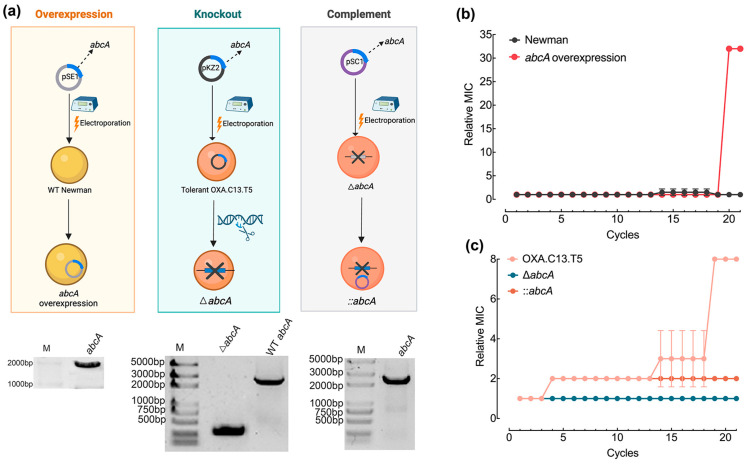
Effect of *abcA* overexpression and deficiency on resistance development. (**a**) Experiment scheme and gel electrophoresis confirmation for constructing *abcA* mutants. (**b**) Relative MIC dynamics for the *abcA*-overexpressing strain (red) versus wildtype Newman (gray). (**c**) Resistance development of *abcA* knockout strain (blue line), tolerant strain (salmon lines), and *abcA* complementary strain (tangerine line). Relative MIC represents the MIC value of each cycle relative to that of the first cycle.

To assess whether elevated *abcA* expression facilitates the transition from tolerance to resistance, the gene was introduced into the wildtype Newman strain to generate an overexpression mutant (Figure 3a). Meanwhile, *abcA* was deleted from the tolerant OXA.C13.T5 background, and a complementation strain was also established (Figure 3a). All constructed mutants underwent 21 cycles of oxacillin exposure at 20× MIC, following the protocol outlined in Figure 1a. *abcA* overexpression enabled the wildtype Newman strain to acquire oxacillin resistance by the 20th cycle, reaching a relative MIC of 30, whereas the control strain remained susceptible throughout (Figure 3b). In contrast, the knockout of *abcA* in the tolerant OXA.C13.T5 background prevented further resistance acquisition, and the strain retained susceptibility (Figure 3c). Additionally, the *abcA* complement failed to restore the development of oxacillin resistance (Figure 3c). These results collectively confirm that increased *abcA* expression facilitated resistance development against oxacillin.

### 3.4. Elevated AbcA Expression Increased the Emergence of Resistance Mutations

**Figure 4:**
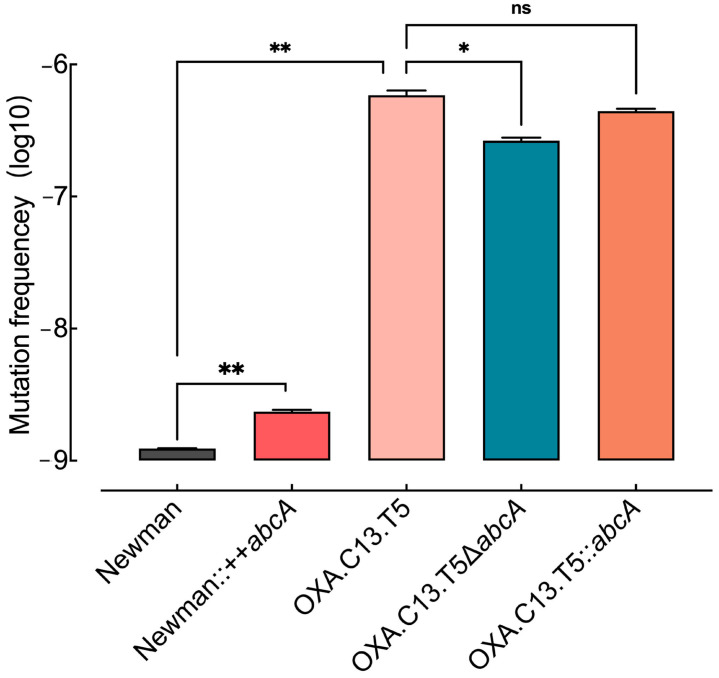
Random mutation rates of either *abcA* overexpression, knockout, and complement mutants. Random mutation rates were evaluated across *abcA*-related mutants, including the wildtype, overexpression, knockout, and complemented strains, as well as the tolerant OXA.C13.T5. Statistical comparisons are based on ≥3 independent experiments. Error bars indicate standard deviation. ns not significant, * *p* < 0.05, ** *p* < 0.01.

To understand how the increased *abcA* expression facilitated the development of resistance against oxacillin, we hypothesized that increased mutation rates might promote emergence of resistance mutation. To test this hypothesis, spontaneous mutation frequencies were assessed across different *abcA* mutants. As illustrated in Figure 4, the *abcA* overexpression strain exhibited a markedly higher mutation frequency than the control, whereas the knockout of *abcA* led to a notable decrease relative to the tolerant background. The reintroduction of *abcA* restored the mutation rate to levels comparable with the parental tolerant strain. These findings suggest that the upregulation of *abcA* promotes mutational emergence, thereby increasing the likelihood of resistance-conferring variants.

### 3.5. Elevated AbcA Expression Increased Bacterial Survival Ability

**Figure 5:**
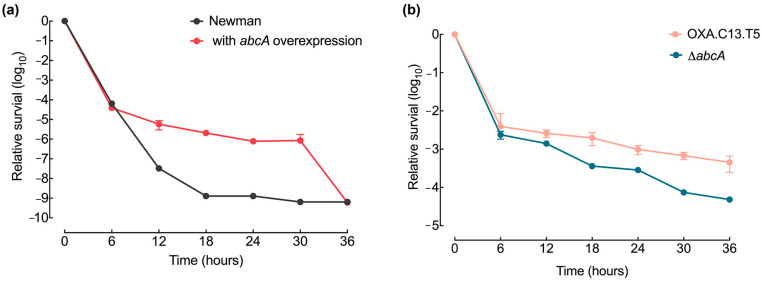
Effect of *abcA* mutants on survival ability. (**a**) Time-dependent survival profile of the *abcA*-overexpressing strain (red), compared with the control Newman strain (gray). CFU values were normalized to initial values. (**b**) Survival curves of the tolerant OXA.C13.T5 (salmon) and *abcA* knockout mutant (blue) during exposure to oxacillin at 20× MIC. Data represent mean ± standard deviation from a minimum of three independent experiments.

Even if resistance mutation emerged, they would disappear without robust survival capacities. To assess whether elevated *abcA* expression enhances bacterial persistence, we tracked the viability of the *abcA* mutant strains during a 36 h exposure to oxacillin at 20× MIC. The overexpression of *abcA* led to a higher survival ability than the wildtype strain under the oxacillin treatment (Figure 5a), while the *abcA* knockout strain showed a decrease in relative survival compared to the control strain OXA.C13.T5 (Figure 5b). These results demonstrate that elevated *abcA* expression increased survival capacities, ensuring the spread of the emerging resistance mutations throughout the population.

## 4. Discussion

Tolerance (including persistence) and resistance are distinct strategies by which bacteria withstand antibiotic stress. Both strategies reduce antibiotic efficacy, but how tolerance promotes resistance remains incompletely understood. Levin-Reisman et al. [32] showed that tolerance can synergize with resistance mutations to enhance survival, yet the progression from tolerance to resistance is still poorly characterized. In this study, we explored this transition, highlighting the role of the efflux pump *abcA*. The tolerant strain OXA.C13.T5 acquired oxacillin resistance earlier than the wildtype Newman (Figure 1), suggesting that tolerance background accelerates resistance development. This observation was consistent across *S. aureus* and *E. coli* [11,33] and under different treatment conditions, including intermittent β-lactam exposure [19] and drug combinations [34], where tolerance mutations preceded resistance acquisition. These findings support a broader model in which tolerance facilitates resistance evolution, posing a clinical threat by potentially leading to the ultimate failure of antibiotics and underscoring the need to disrupt this trajectory through targeted interventions.

Although tolerance has been linked to the emergence of resistance, the molecular drivers underlying this transition remain unclear. Our findings identify the efflux pump *abcA* as a key factor mediating this progression. AbcA transporters are implicated in diverse physiological roles across organisms, including multidrug resistance in cancer [35,36]. In *S. aureus*, AbcA contributes both to β-lactam resistance and virulence via cytolytic toxin secretion [24,27]. Here, we propose a the following third role: facilitating the shift from tolerance—a major cause of recurrent infection—to full resistance, thereby compromising antibiotic efficacy. We observed significantly elevated *abcA* expression in tolerant strains (OXA.C13.T5, IMI.C9.T5, and FLUC.C20.T5) compared to the wildtype strain (Figure 2b), consistent with the findings of Pu et al. (2016), where there was increased efflux gene expression in *E. coli* persisters [12]. These findings support a role for efflux systems in establishing tolerance under β-lactam exposure. Furthermore, previous studies on *norA* and *tolC* have shown that the deletion of efflux components reduces tolerance [16,30], while *norA* upregulation accelerates ciprofloxacin resistance [16,37]. Based on these observations, we hypothesized that *abcA* facilitates the tolerance-to-resistance transition. This was supported by our data showing faster resistance emergence in *abcA*-overexpressing strains and the complete failure of resistance acquisition in *abcA* knockout strains (Figure 3b,c). Parallel patterns observed in *norA* mutants suggest that efflux-mediated transitions may represent a broader adaptive mechanism across efflux families.

Our findings indicate that tolerance promotes the evolution of antibiotic resistance by enhancing both survival and mutation rates. Repeated oxacillin exposure in the wildtype Newman strain led to tolerance but not resistance (Figure 1b). The whole-genome sequencing of the tolerant OXA.C13.T5 strain revealed a single mutation in *gdpp*, unrelated to survival or mutagenesis (Table S1). Functional comparisons showed that *abcA* overexpression significantly increased mutation frequency and survival under oxacillin treatment (Figure 4 and Figure 5), while knockout impaired both. These results suggest that *abcA* facilitates resistance evolution by simultaneously improving the likelihood of resistance mutations and supporting the survival of emerging mutants. Notably, although *abcA* complementation restored mutation frequency, it did not result in resistance acquisition. This indicates that while elevated mutability increases the chance of resistance mutations, it is insufficient to establish the resistance phenotype. In particular, plasmid-based *abcA* expression may lack the stress-inducible regulation necessary for resistance development and fixation under intermittent antibiotic exposure. Mechanistically, our results align with Windels et al.’s model in *E. coli*, which proposed that enhanced survival and mutation jointly drive resistance [17]. We extend this concept by demonstrating that the *abcA*-mediated increase in both traits enables the transition from tolerance to resistance. Similar trends observed with *norA* [16] and elevated *abcA* expression in multiple tolerant strains (Figure 2b) suggest a broader role for efflux pumps. Furthermore, our intermittent antibiotic exposure model reflects clinical treatment regimens [38], supporting the translational relevance of *abcA*-driven adaptation.

To our knowledge, this study is the first to demonstrate that *abcA* plays a direct role in promoting oxacillin resistance from a tolerant state. While prior research has broadly linked tolerance to resistance, our findings uniquely identify AbcA as a mechanistic driver of this transition. This insight presents AbcA as a potential therapeutic target. For example, combining antibiotics with AbcA inhibitors could reduce resistance emergence and improve treatment efficacy. Such a strategy may enable both simultaneous bacterial clearance and suppression of resistance development. These results also underscore the importance of monitoring efflux pump activity as part of resistance management in clinical settings and aligning with the antibiotic stewardship principles that advocate for informed drug use. To model clinical exposure, we employed intermittent exposure to 20× MIC oxacillin based on the established in vitro evolution protocols [11,19]. Although 20× MIC exceeds the standard plasma levels, such concentrations are achievable in local administration settings. For instance, cefazolin irrigation during surgery produces wound site concentrations > 4000 µg/mL [39], and the intramammary infusion of cloxacillin can maintain concentrations > 30 µg/mL in the milk for days [40]. These local exposures impose strong selective pressure, supporting the clinical relevance of our experimental model. Despite the robustness of our in vitro findings, limitations remain. The simplified laboratory context cannot fully replicate host environments, and further in vivo validation is needed. Future research should also explore whether other efflux systems share this tolerance-to-resistance bridging function. Understanding such mechanisms may inform broader strategies to delay or prevent resistance evolution.

## 5. Conclusions

In conclusion, our data map an evolutionary pathway from tolerance to resistance, demonstrating that the increased expression of efflux pump *abcA* not only accelerated the generation of resistance mutations but also enhanced the survival of resistant mutants. This comprehensive understanding of the evolution from tolerance to resistance is crucial for developing more effective antibiotic treatment strategies for combating the growing challenge of antibiotic inefficiency due to the emergence of resistance.

## Data Availability

The data are available upon request to the corresponding author.

## Supplementary Materials

The following supporting information can be downloaded at: https://www.mdpi.com/article/10.3390/microorganisms13051140/s1, Table S1: *Staphylococcus aureus* strains used in this study; Table S2: Primers used in this study.

## Abbreviations

The following abbreviations are used in this manuscript:

*S. aureus*	*Staphylococcus aureus*
*E. coli*	*Escherichia coli*
MIC	Minimum Inhibitory Concentration
