# Supplementary material for: Increased Expression of AbcA Efflux Pump Accelerated Resistance Development from Tolerance to Resistance Against Oxacillin in Staphylococcus aureus
Source: Microorganisms. 2025 May 16;13(5):1140. doi: 10.3390/microorganisms13051140 (PMC12113836; doi:10.3390/microorganisms13051140)
Supplement: Supplementary file 1 [file microorganisms-13-01140-s001.zip › microorganisms-3600399-supplementary.pdf]

Supplementary Information

Yu et al. Increased expression of AbcA efflux pump accelerated the rapid resistance development from tolerance to resistance against oxacillin in *Staphylococcus aureus*

Evolutionary adaptation procedure

In our previous research, we successfully obtained four *S. aureus* strains with antibiotic tolerance (detailed in Supplementary Table S1), utilizing a structured evolutionary adaptation experiment. This adaptation procedure comprised three key steps: an exposure phase, an antibiotic clearance phase, and regrowth and analysis. These tolerant strains were used in our current investigation into the role of the efflux pump gene *abcA* in the progression of antibiotic resistance.

Exposure Phase: Overnight cultures of *S. aureus* (0.5 ml, ~2×10^9 CFU/ml) were diluted into 50 ml of TSB supplemented with oxacillin, imipenem, flucloxacillin, or meropenem at 20x their minimum inhibitory concentrations (MIC). These cultures were incubated at 37°C with a shaking speed of 180 rpm for the duration of 5 hours.

Antibiotic Clearance Phase: Following exposure, the cultures underwent a dual washing process using Phosphate Buffered Saline (PBS), each involving centrifugation at 1,500 g for 20 minutes to remove residual antibiotics.

Regrowth and Analysis: After washing, the bacteria were resuspended in 1 ml of fresh TSB with selective antibiotics and incubated overnight at 37°C. This process was repeated for 9 to 21 cycles, with the MIC evaluated after each cycle. Strains demonstrating tolerance prior to the onset of resistance were isolated and purified. After confirming the tolerance phenotypes of these strains, genome sequencing was performed. The mutations associated with the tolerance

phenotypes were identified by restoring the mutated genes to measure tolerance alterations and are listed in Supplementary Table S1. Four tolerant strains possessed mutations in genes such as *gdpp* encoding c-di-AMP phosphodiesterase for OXA.C13.T5, *pth* encoding peptidyl-tRNA hydrolase for IMI.C9.T5, *545* encoding unknown 545 for FLUC.C20.T5 and *map* encoding MHC class II analog protein for FLUC.C20.T5.

Table S1 *Staphylococcus aureus* strains used in this study

Strain	Gene	Annotation	Mutation	Amino acid substitution	Phenotype
**Newman**	—	—	—	—	Susceptible to antibiotics
**OXA.C13.T5**	*gdpp*	c-di-AMP phosphodiesterase	G<->A	G282D (GGC<->GAC)	Tolerant to oxacillin
**IMI.C9.T5**	*pth*	Peptidyl-tRNA hydrolase	A<->G	K127E (AAA<->GAA)	Tolerance to imipenem
**FLUC.C20.T5**	*545* (new)	Unknown	G< >A	M31I (ATG<->ATA)	Tolerant to flucloxacillin
**MER.C19.T5**	*map*	MHC class II analog protein	C< >A	E323- (TTC<->TTA)	Tolerant to meropenem

Note: The first part of the tolerant strain name denotes the antibiotic type of treatment, the middle part means cycles for antibiotic exposure in the previously evolutionary protocol, while the last part indicates the duration time of the antibiotic (Ta = 5 h.). The whole genome sequence revealed mutations in gene *gdpp*, *pth*, 545 (unknown), and *map* whose functions were not related to efflux pumps. These mutations were identified to be responsible for tolerance phenotype by restoring isogenic genes (unpublished data).

Mutant-construction procedure

Vectors (pSE1, pKZ2, and pSC1) were first linearized using EcoRI and KpnI enzymes and purified using the Universal DNA Purification Kit. The *abcA* gene was PCR-amplified from the genomic DNA of the Newman strain, with primers designed for Gibson assembly (Table S2). The linearized vectors and amplified *abcA* gene were assembled using the Peasy-Basic Seamless Cloning and Assembly Kit. Assembled vectors were transformed into *E. coli* DH5α competent cells. Clones were selected on LB agar plates containing carbenicillin (100 mg/L) and confirmed via PCR and Sanger sequencing. Subsequently, these vectors were introduced into IM08B and targeted *S. aureus* strains through electroporation. *S. aureus* transformants were selected on TSB agar plates containing chloramphenicol (10 mg/L) and further validated by PCR and Sanger sequencing.

Table S2 Primers used in this study

Name	Sequence 5'>3'
**Primes for quantifying expression of efflux pumps**	
RT-16S-F	GCTCGTGTCGTGAGATGTTGG
RT-16S-R	TTTCGCTGCCCTTTGTATTGT
RT-*abcA*-F	AACCGACAGAAGCTCTTGAAG
RT-*abcA*-R	CCTTGTGGGATTTGGAACGAC
**Primers for overexpression of *abcA***	
OE-*abcA*-F	gtagtcttatataaggaggataatgggtaccATGAAACGAGAAAATCCATTGTTTTTC
OE-*abcA*-R	ccagtgtgatggatatctgcagaattcTTAATCTGTTAATTTTTGAGACACTACAAAG
CLF	TATCCTAACAGCACAAGAGC
M13F	GTAAAACGACGGCCAGT
**Primers for constructing *abcA* knockouts**	
KO-*abcA*-1	TGAGCCTCGGAACCGGTACCCTTTGTTGGTGCAAATGTAC
KO-*abcA*-2	GCTCCAAGCTTACTTATATATATAAAAAGTTTTACCTCTTCTGTT
KO-*abcA*-3	CAAACAGAAGAGGTAAAACTTTTTATATATATAAGTAAGCTTGG
KO-*abcA*-4	CCGGCGGCCGCTCGGAATTCCACTTACTGGATTCAACAAG
**Primers for constructing *abcA* complements**	
CL-*abcA*-F	GCAAAATGGTCGACGGTACAAAGCGTTAATCTTCCCTTTTCC
CL-*abcA*-R	CGCCAGTGTGATGGATATCTGCAGTTAATCTGTTAATTTTTGAGAC

Determination of minimum inhibitory concentrations (MIC)

The MICs for *S. aureus* strains during the evolution were determined using the broth microdilution method in 96-well microtiter plates. This protocol began with the suspension of three morphologically consistent colonies from each strain in TSB for overnight incubation. The overnight cultures were adjusted to an optical density at 600 nm (OD600) of 0.16-0.18 for

approximately 1 × 10^8 cells/ml. The standardized inoculum was diluted 40-fold in Mueller–Hinton II broth (Sigma-Aldrich Co., St. Louis, MO, United States). A range of antibiotic concentrations was prepared in the broth, creating a gradient from 64 mg/L to 0.03125 mg/L. After 24 h of incubation at 37°C, the MIC was defined as the lowest concentration of antibiotic that visibly inhibited bacterial growth. Interpretation of oxacillin susceptibility in *S. aureus* was based on the Clinical and Laboratory Standards Institute (CLSI) MIC Interpretive Criteria, categorizing susceptibility at ≤ mg/L and resistance at ≥ 4 mg/L. The MIC values for oxacillin in both the parent Newman strain and the evolved OXA.C13 strain were 0.5 mg/L.

Quantitative real-time PCR analysis of efflux pump expression

Frozen stocks of the tolerant strains and the wildtype Newman were diluted 1:1,000 into fresh TSB medium for overnight culture. For each group, 5 μL of the overnight culture was inoculated into 5 mL TSB which contained 20× MIC concentrations of respective antibiotics, mimicking conditions in the prior evolutionary experiments. After five hours of shaking at 180 rpm in a 37°C incubator, tolerant and wildtype bacterial cells were collected by centrifuging for 5 minutes at 5,000 × g. Total RNA was extracted using the E.Z.N.A.®HP Total RNA Kit (Omega, Norcross, GA, USA), with procedures adapted for Gram-positive bacteria. RNA quality was verified by absorbance ratios at 230, 260, and 280 nm and agarose gel electrophoresis. Then, the extracted RNA was reverse transcribed into cDNA using the TransScript One-step gDNA Removal and cDNA Synthesis SuperMix (TransGen, Beijing, China). The expressional levels of pump genes were determined by quantitative PCR in a 25-μl TransStart Tip Green qPCR supermix (TransGen) using 2 μl of diluted cDNA as the template. The expression of pumps was calculated by the $2^{-\Delta\Delta CT}$ method, with 16S rRNA as a reference. The calculated pump expression of tolerant strains was log2 normalized relative to the mean

expression level of the wildtype strain Newman. Mean fold change values were equivalent to the normalized expression ratio. All the primers for qPCR are listed in Table S2.
